# Visual Outcomes of Adding Erythropoietin to Methylprednisolone for Treatment of Retrobulbar Optic Neuritis

**DOI:** 10.18502/jovr.v14i3.4786

**Published:** 2019-07-18

**Authors:** Mostafa Soltan Sanjari, Farzad Pakdel, Fatemeh Moosavi, Niloofar Pirmarzdashti, Marzieh Nojomi, Anoosheh Haghighi, Masih Hashemi, Mohsen Bahmani Kashkouli

**Affiliations:** ^1^ Ophthalmology Department, Eye Research Center, Rassoul Akram Hospital, Iran University of Medical Sciences, Tehran, Iran; ^2^ Ophthalmology Department, Eye Research Center, Farabi Eye Hospital, Tehran University of Medical Sciences, Tehran, Iran; ^3^ Department of Community Medicine, Iran University of Medical Sciences, Tehran, Iran; ^4^ Internal Medicine Department, Rassoul Akram Hospital, Iran University of Medical Sciences, Tehran, Iran

**Keywords:** Contrast Sensitivity, Erythropoietin, Optic Neuritis, Optic Neuroprotection, Optic Nerve Regeneration, Visual Acuity, Visual Function

## Abstract

**Purpose:**

To compare the short-term visual function results and safety of erythropoietin as an add-on to the standard corticosteroid therapy in retrobulbar optic neuritis (RON).

**Methods:**

In this prospective pilot study, adult patients with isolated RON with less than 10 days of onset were enrolled. Patients were consecutively assigned to standard intravenous methylprednisolone treatment either in combination with intravenous erythropoietin (20,000 units/day for three days) (group-1) or intravenous methylprednisolone alone (group-2). Primary outcome measure was best-corrected visual acuity (BCVA), which was assessed up to 120 days from the day the treatment was begun. Systemic evaluations were performed during and after treatment.

**Results:**

Sixty-two patients with RON (mean age = 26.6 ± 5.77 years; range = 18–40 years) were enrolled into the study (group-1, n = 35; group-2, n = 27). BCVA three months after the treatment was 0.19 ± 0.55 logMAR and 0.11 ± 0.32 logMAR in group-1 and group-2, respectively (95% CI: -0.61 - 0.16; P = 0.62). Change in BCVA after three months was 2.84 ± 3.49 logMAR in group-1 and 2.46 ± 1.40 logMAR in group-2 (95% CI: -0.93-1.91; P = 0.57). Pace of recovery was not significantly different between the groups. No complications were detected among patients.

**Conclusion:**

Intravenous erythropoietin as an add-on did not significantly improve the visual outcome in terms of visual acuity, visual field, and contrast sensitivity compared to traditional intravenous corticosteroid. This pilot study supports the safety profile of intravenous human recombinant erythropoietin, and it may help formulate future investigations with a larger sample size.

## INTRODUCTION

Acute optic neuritis is an inflammatory, demyelinating disorder. It is the most common optic neuropathy affecting young adults.^[1]^ Main pathologic features include microglial and T cell infiltration, edema, myelin breakdown, axonal and neuronal degeneration, and astrogliosis of the optic nerve.^[2,3,4]^ Following an episode of optic neuritis, the optic nerve undergoes atrophy.^[5]^ Although, in most patients, visual acuity recovers rapidly following an episode of acute optic neuritis, significant number of patients suffer from disturbances of visual functions such as best-corrected visual acuity (BCVA), contrast sensitivity (CS), color vision, and visual field.^[5,6,7]^ In an optic neuritis treatment trial (ONTT) study, which involved long-term follow-up, 26% of the patients showed a visual acuity that was lower than 20/20 in the affected eye, and 33% showed abnormal CS and visual field.^[7]^ Methylprednisolone pulse therapy is the standard treatment for acute optic neuritis. Although it accelerates visual recovery, it does not influence visual outcome, lesion length, or atrophy of the optic nerve.^[4,7,8]^ Furthermore, the safety of high dose corticosteroid treatment on retinal ganglion cell (RGC) survival is debated. An experimental study on an optic neuritis model showed that methylprednisolone increased RGC degeneration by inhibiting an endogenous neurotrophin-dependent pathway.^[9]^

In addition to the treatment using anti-inflammatory and immunomodulatory agents, neuroprotection and neuroregeneration are other intriguing treatment strategies. Erythropoietin has shown neuroprotective and neuroregenerative properties. Recent experimental and clinical studies support the efficacy of erythropoietin in various clinical optic nerve disorders including traumatic optic neuropathy,^[10,11]^ ischemic optic neuropathy,^[12]^ toxic optic neuropathy,^[13]^ and optic neuritis.^[14,15]^ In a recent study, patients treated with a combination of corticosteroid and erythropoietin were found to have higher retinal nerve fiber layer (RNFL) thickness and optic nerve diameter.^[14]^ In the current study, we aimed to compare different optic nerve function parameters in patients with isolated retrobulbar optic neuritis (RON), who were subjected to high dose corticosteroid treatment either in combination with erythropoietin or alone.

##  METHODS

This was a prospective pilot study conducted in a tertiary university-based referral hospital. The ethics committee of eye research center affiliated to Rassoul Akram Hospital, Iran University of Medical Sciences approved this study. The purpose of the study and its possible outcomes and adverse events were explained to all participants, and written informed consents were obtained. The study was conducted according to the tenets of Helsinki Declaration. From November 2010 to March 2013, patients with acute visual loss and preliminary diagnosis of RON, who were referred from general and neuro-ophthalmology clinics, were evaluated. The eligibility criteria were mainly based on ONTT [Table 1].

**Table 1:** Eligibility criteria


**Inclusion Criteria**	**Exclusion Criteria**
Acute visual symptoms	Pregnancy
Normal optic disc and fundoscopic exam with 78 D lens	Breastfeeding
Relative afferent pupillary defect	Clinically definite MS
Visual field defect in the affected eye	Hyperopia > 3 diopter, myopia > -5.0 diopter, irregular astigmatism
Age range of 18 years to 40 years	Elevated blood pressure (systolic > 140 mmHg, Diastolic > 90 mmHg)
No previous episodes of optic neuritis in the affected eye	History of thrombi-embolic events
No previous congenital or acquired ophthalmological morbidity hindering the visual functions	Malignancy
No previous corticosteroid or erythropoietin treatment for optic neuritis	Seizures
No systemic disease	History of collagen vascular disease
Best-corrected visual acuity ≤ 20/40	Sarcoidosis
Duration of symptoms less than 10 days	Graves' disease
	Heavy cigarette smoking
	History or clinical findings for severe ophthalmic diseases such as central retinal vein occlusion
	Any hereditary or acquired macular disease
	History of any intraocular or keratorefractive surgery
	Any abnormal findings in orbital or brain MRI suggestive of decreased visual function
	
	
MRI, magnetic resonance imaging; MS, multiple sclerosis	

All patients underwent a comprehensive ophthalmologic evaluation, which included assessment of detailed history, BCVA (with a standard Landolt C chart in a single room), relative afferent pupillary response, color vision (with Ishihara plates), extra-ocular movements, and intra-ocular pressure (by application tonometry), in addition to dilated funduscopic examination (with 78 D lens) and anterior segment examination (with slit lamp). Diagnosis of isolated RON was confirmed by an expert neuro-ophthalmologist.

All patients were hospitalized. Complete blood cell count, hematocrit, aspartate transaminase (AST), alanine transaminase (ALT), alkaline phosphatase, blood urea nitrogen, blood creatinine, blood sugar, and C-reactive protein, in addition to serum sodium, calcium, phosphorus, and magnesium were evaluated. In the case of abnormal lab data, treatment was stopped and an internal medicine specialist was consulted.

Patients were subsequently assigned into one of the two treatment groups after obtaining informed consent; those receiving intravenous human recombinant erythropoietin and methylprednisolone for three successive days were assigned to group-1 and those receiving only intravenous methylprednisolone for three successive days were assigned to group-2. Patients in both groups received oral prednisolone 1 mg/kg/day for additional 11 days after the intravenous treatment.

All patients received 250 mg intravenous methylprednisolone every six hours. In addition, 20,000 international units of intravenous recombinant human erythropoietin (PDpoetin, Pooyesh Darou Pharmaceutical Co., Tehran, Iran) was infused into 200 ml normal saline in group-1. Patients in group-2 received 200 ml normal saline per day as placebo. Systolic and diastolic blood pressures were checked every 15 minutes during erythropoietin infusion. The patients were excluded from the study if they met the exclusion criteria.

Inclusion, group assignment, and management process were directed by an expert ophthalmologist (FP). Follow-up examinations were performed using the same charts and instruments by a senior ophthalmology resident (FM). Both neuro-ophthalmologist and examiner were blind to the treatment protocol.

Primary outcome measure was BCVA. Visual acuity was measured by standard Landolt C acuity chart after best spectacle correction. Color vision was checked with 15 Ishihara plates if visual acuity was 20/160 (0.9 logMAR) or better. Contrast sensitivity (CS) was checked using a YANG vision tester (SIFI MEDTECH group Co., Lavinaio, Italy) at a distance of 3 m and spatial frequency of 6-cycle/degree (c/d) at 30–70 foot-lambert illumination. All visual functions were assessed before treatment and at days 1, 2, 3, 14, 30, 60, and 120 after treatment. Visual field was evaluated with Humphrey 750 using C-24-2 SITA-standard strategy (target size = 3, white target) on days 0, 14, 30, 60, and 120 if BCVA was 20/200 (logMAR = 1.0) or better. Hemoglobin, hematocrit, and platelet count were checked on days 0, 30, and 60 after treatment.

A probability value of less than 0.05 was considered significant for all statistical tests. The independent sample *t*-test was used for the comparison of normal numeric parameters. Kaplan–Meier survival analysis was performed to estimate time to event; the cut-off point for BCVA was logMAR ≤ 0 and that for CS was ≥70.^[15]^ Statistical analyses were carried out with the Statistical Package for Social Sciences (SPSS Package, version 22, IBM Corporation, Armonk, New York, USA).

##  RESULTS

We evaluated 102 patients with acute isolated RON. Thirty-five patients received corticosteroid treatment in combination with erythropoietin (group-1) and twenty-seven subjects received corticosteroid treatment alone (group-2). Forty cases were excluded because they did not fulfill the inclusion criteria or because they met the exclusion criteria [Table 1].

The mean age of the included patients was 26.6 ± 5.77 (range = 18–40) years. Of 62 patients, 43 were female (69.35%). Time delay in treatment (interval between the onset of symptoms and initiation of treatment) in both groups did not differ significantly. Demographic data are shown in Table 2.

**Table 2:** Demographics of patients with retrobulbar optic neuritis


	**Group-1 **	**Group-2 **	**** ***P*** ** value**
Age (mean ± SD; years)	25.54 ± 5.943	27.70 ± 5.587	0.14
Sex (F:M)	29 (70.7%): 12 (29.3)	25 (64.1%):14 (35.9%)	0.63
Time interval ± SD (days)*	5.91 ± 2.66	5.29 ± 2.34	0.33
	
	
*Time interval between onset of visual symptoms and starting protocol treatment			
Group-1 = Combined erythropoietin and methylprednisolone treatment			
Group-2 = Methylprednisolone treatment			
F, female; M, male; SD, standard deviation			

**Figure 1:**
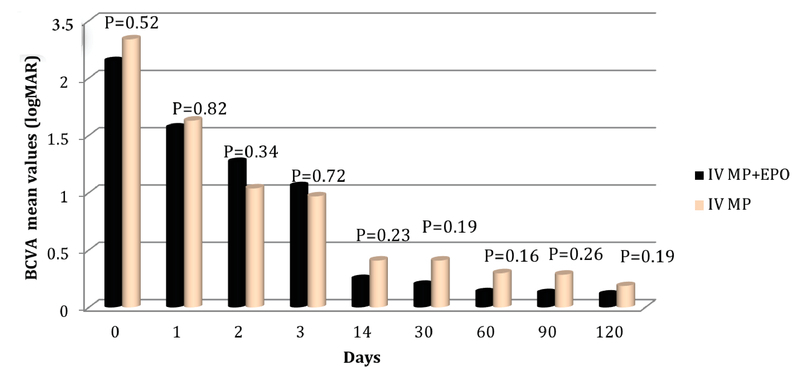
Best-corrected visual acuity (BCVA) at different time intervals among patients with retrobulbar optic neuritis in the two treatment groups. IV MP, intravenous methylprednisolone; IV MP + EPO, intravenous methylprednisolone in combination with intravenous erythropoietin.

At baseline, there was no statistically significant difference in mean visual acuity (represented in logMAR) between group-2 (2.35 ± 1.25) and group-1 (2.15 ± 1.18) (*P* = 0.52). Three months after treatment, BCVA of 20/20 or better (logMAR ≤ 0) was achieved in 48 (66.1%) patients, among which 29 (71.4%) patients belonged to group-1 and 19 (59.3%) to group-2. BCVA at three months follow-up time-point was found to be 0.11 ± 0.32 logMAR and 0.19 ± 0.55 logMAR in group-2 and group-1 (*P* = 0.51), respectively [Figure 1].

The pace of recovery was not different between the two groups. BCVA of 20/20 or better (log MAR ≤ 0) was achieved in 34.52 days after treatment in group-1 and in 41.12 days after treatment in group-2 (*P *= 0.59). Change in BCVA after three months was 2.84 ± 3.49 logMAR in group-1 and 2.46 ±1.40 in group-2 (*P* = 0.632), compared to pre-treatment baseline values.

**Figure 2:**
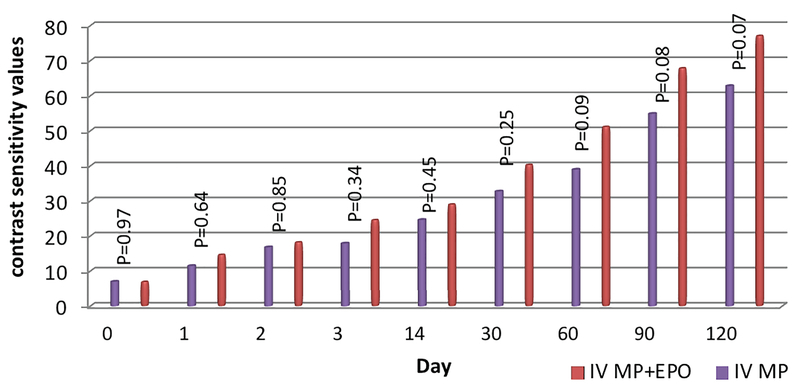
Comparison of contrast sensitivity in patients with retrobulbar optic neuritis in the two treatment groups. IV MP, intravenous methylprednisolone; IV MP + EPO, intravenous methylprednisolone in combination with intravenous erythropoietin.

Baseline CS (represented in 6 C/D) was 6.85 ± 9.1 in group-1 and 6.98 ± 7.36 in group-2. Improvement of CS (cut-off point > 70) was achieved after three months in 25 (40.3%) patients with RON, among which 18 (51.4%) patients belonged to group-1 and 7 (25.9%) to group-2. CS was not significantly different between the two groups. Cut-off point of CS (> 70) improved in 73.33 days (range = 58.13–88.53) in group-1 and in 85.71 days (range = 61.95–109.47) in group-2 [Figure 2]. The change in CS after three months was observed to be 65.78 ± 32.32 in group-1 and 51.09 ± 33.92 in group-2 (*P* = 0.078).

**Figure 3:**
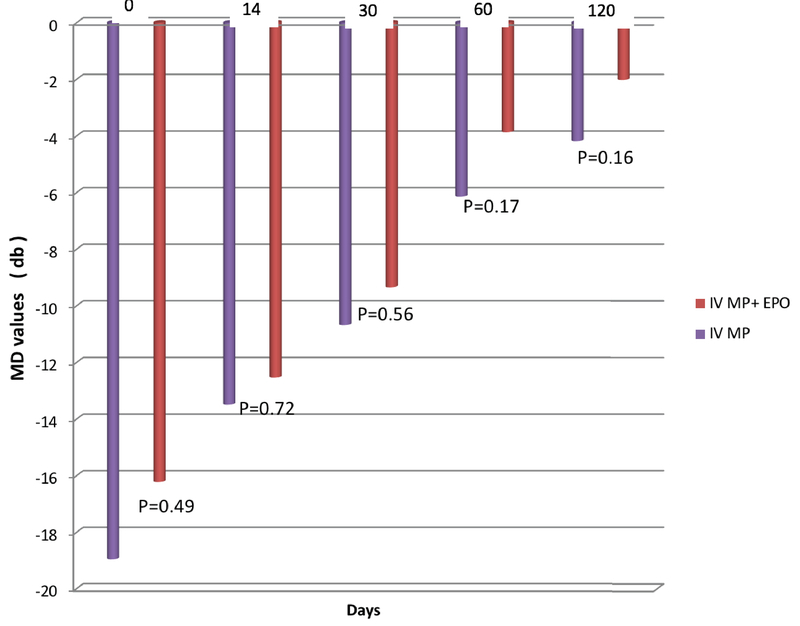
Mean deviation (MD) in patients with retrobulbar optic neuritis in the two treatment groups. IV MP, intravenous methylprednisolone; IV MP + EPO, intravenous methylprednisolone in combination with intravenous erythropoietin.

Analysis of mean deviation (MD) showed insignificant differences among participants in group-1 and group-2 after three months. Pattern standard deviation (PSD) showed no significant differences between the two groups except on day 60 (*P* = 0.002) [Figure 3].

Mean baseline values of color vision test were 2.3 ± 2.06 and 3.37 ± 2.72 in group-1 and group-2 (*P* = 0.377), respectively. Four months after treatment, mean color vision values of patients in group-1 and group-2 were 11.96 ± 4.49 and 12.94 ± 2.68, respectively (*P* = 0.338).

There was no significant difference in the platelet count change at days 30 and 60 after treatment in both groups. Mean hematocrit change in the male and female patients of group-1 and group-2 after 30 and 60 days of treatment was statistically insignificant. We observed transient increase in systolic blood pressure in a 35-year-old patient during intravenous infusion of erythropoietin. No further increase in blood pressure was recorded during follow-up. During treatment and follow-up period, no remarkable complications were detected among patients.

##  DISCUSSION

In this pilot study, we have compared short-term visual function results of intravenous methylprednisolone with and without erythropoietin as an add-on in patients with acute RON.

Results of ONTT showed that corticosteroids could accelerate visual recovery.^[4]^ However, in about one-third of these patients, residual disturbances in various aspects of visual functions, including central visual acuity, CS, and visual field can persist.^[7]^ Optic neuritis is associated with RGC loss and RNFL thinning.^[16]^ Axonal loss in the RNFL has consistently been demonstrated following optic neuritis,^[16]^ and after an episode of optic neuritis, 74% of the patients show thinning of RNFL, denoting axonal damage, and RGC loss in addition to their correlation to visual function deficit.^[17]^ Furthermore, high dose corticosteroid therapy possibly increases RGC apoptosis following optic neuritis; this is an issue of concern.^[5]^ These findings signify permanent structural and functional deficits in the visual system after an episode of optic neuritis and, thus, justify the use of neuroprotective and regenerative treatment options.

Erythropoietin, which has long been known as a hematopoietic cytokine, can protect neurons from apoptosis^[18,19,20]^ and show protective effects in experimental models of mechanical optic nerve trauma,^[21]^ inflammation,^[22,23]^ cerebral and retinal ischemia,^[23]^ and oxidative stress,^[24]^ in addition to animal model of optic neuritis.^[9,25]^ Clinical studies over the recent decade have also supported the use of erythropoietin as a neuroprotective and neuroregenerative agent in certain acquired optic neuropathies such as traumatic,${}^{\left[10,11\right]}$ ischemic,^[12]^ demyelinative, inflammatory,^[14]^ and toxic^[13]^ optic neuropathies.

One study on experimental optic neuritis model suggested that both neuronal and axonal protection, in functional and structural aspects, are most effective when combined erythropoietin and methylprednisolone treatment regimen was commenced. Isolated neuronal or axonal protection, without clinical benefit, was achieved under monotherapy with either erythropoietin or methylprednisolone.^[25,26]^

Suhs et al demonstrated lower RNFL thinning and lower values of visual evoked potential parameters in patients with optic neuritis when they received 33,000 units/day of intravenous erythropoietin for three days as an add-on therapy to methylprednisolone compared to those who received only methylprednisolone.^[14]^ The aforementioned studies favored the structural and physiological effects of erythropoietin in treating optic neuritis. However, our study could not show a significant superior effect of the combined intravenous treatment (erythropoietin along with methylprednisolone) in terms of the extent and rapidity of recovery of CS, BCVA, and visual field parameters.

All our patients experienced partial or complete recovery of BCVA after three months. Recovery of CS, visual acuity, visual field, and color vision is characteristic after an episode of optic neuritis; the magnitude of the recovery of the aforementioned visual functions in the current study is comparable to other studies.^[9]^

Although we observed some trends that point toward the achievement of a better visual function in erythropoietin-treated patients, changes in functional parameters were not significantly different between the treatment groups. Furthermore, Suchs et al also did not observe any significant difference in visual acuity in patients who were administered erythropoietin as an add-on to methylprednisolone despite higher RNFL thickness in optical coherence tomography (OCT) measurements.^[14]^ Such discrepancy may be explained at least in part by the fact that papillomacular bundle, rather than mean peripapillary nerve fiber layer thickness, subserves the fovea, which is responsible for the central visual acuity.

CS is a more sensitive visual function than visual acuity. It remains impaired in 33% of the patients, years after acute optic neuritis.^[7]^

We did assess CS, which has recently been reported to detect even subtle visual impairment.^[15]^ During the final follow-up evaluation of patients, improved CS (> 70) was observed in 25 (40.3%) patients, of whom 18 (51.4%) belonged to group-1 and 7 (25.9%) to group-2.

The major complications that were reported with erythropoietin include polycythemia, thrombo-embolic events,^[27]^ hypertensive reactions,^[27]^ and pure red cell aplasia.^[28]^ We did not observe any significant change in hematocrit, platelet count, or any other clinical parameters that can be classified as adverse events. Blood pressure of the participants in both groups remained stable during the treatment period and thereafter, it did not differ significantly between the treatment groups. However, there was an exception. One participant who had received combined erythropoietin and methylprednisolone treatment experienced a mild and transient elevation in systolic blood pressure, which returned to baseline level after the infusion was put on hold for 15 minutes; no further changes were observed after restarting the infusion.

Limitations of this pilot study included relatively small sample size and lack of randomization. Authors cannot ignore one other possible reason for the absence of difference between the two groups, that is, the suppression of the neuroprotective effect of erythropoietin by concurrent corticosteroid therapy. In the future, randomized clinical trials with larger sample size may reveal statistically significant differences in the visual outcome after erythropoietin treatment. For future studies, our calculation shows that each group should have a sample size of 37 subjects to detect a difference of at least 0.2 logMAR and to have 80% power. Structural data such as that obtained through OCT could provide useful complementary data, and it should be regarded in future studies. Furthermore, follow-up time was short. Longer follow-up periods are needed to justify any possible visual benefits in long-term scales in patients with optic neuritis. This may justify future studies with larger sample size and different erythropoietin doses.

In conclusion, we found that adding intravenous human recombinant erythropoietin could not statistically improve short-term visual function results compared to systemic high dose methylprednisolone treatment. Furthermore, this study supports the safety profile of intravenous human recombinant erythropoietin.

##  Financial Support and Sponsorship

Nil.

## Conflicts of Interest

There are no conflicts of interest.
